# BLR1 and FCGR1A transcripts in peripheral blood associate with the extent of intrathoracic tuberculosis in children and predict treatment outcome

**DOI:** 10.1038/srep38841

**Published:** 2016-12-12

**Authors:** Synne Jenum, Rasmus Bakken, S. Dhanasekaran, Aparna Mukherjee, Rakesh Lodha, Sarman Singh, Varinder Singh, Marielle C. Haks, Tom H. M. Ottenhoff, S. K. Kabra, T. Mark Doherty, Christian Ritz, Harleen M. S. Grewal

**Affiliations:** 1 Department of Clinical Science, Faculty of Medicine and Dentistry, University of Bergen, Bergen, Norway; 2 Department of Infectious Diseases, Oslo University Hospital, Oslo, Norway; 3 Department of Pediatrics, All India Institute of Medical Sciences, New Delhi, India; 4 Division of Clinical Microbiology & Molecular Medicine, Department of Laboratory Medicine, All India Institute of Medical Sciences, New Delhi, India; 5 Department of Pediatrics, Kalawati Saran Children Hospital, New Delhi, India; 6 Department of Infectious Diseases Group, Immunology and Immunogenetics of Bacterial Infectious Disease, Leiden University Medical Center, The Netherland; 7 GlaxoSmithKline Vaccines, Warve, Belgium; 8 Department of Nutrition, Exercise and Sports, University of Copenhagen, Denmark; 9 Department of Microbiology, Haukeland university hospital, University of Bergen, N-5021, Norway

## Abstract

Biomarkers reflecting the extent of *Mycobacterium tuberculosis*-induced pathology and normalization during anti-tuberculosis treatment (ATT) would considerably facilitate trials of new treatment regimens and the identification of patients with treatment failure. Therefore, in a cohort of 99 Indian children with intrathoracic tuberculosis (TB), we performed blood transcriptome kinetic analysis during ATT to explore 1) the association between transcriptional biomarkers in whole blood (WB) and the extent of TB disease at diagnosis and treatment outcomes at 2 and 6 months, and 2) the potential of the biomarkers to predict treatment response at 2 and 6 months. We present the first data on the association between transcriptional biomarkers and the extent of TB disease as well as outcome of ATT in children: Expression of three genes down-regulated on ATT (*FCGR1A, FPR1 and MMP9*) exhibited a positive correlation with the extent of TB disease, whereas expression of eight up-regulated genes (*BCL, BLR1, CASP8, CD3E, CD4, CD19, IL7R* and *TGFBR2*) exhibited a negative correlation with the extent of disease. Baseline levels of these transcripts displayed an individual capacity >70% to predict the six-month treatment outcome. In particular, *BLR1* and *FCGR1A* seem to have a potential in monitoring and perhaps tailoring future antituberculosis therapy.

The emerging epidemic of multi-drug resistant tuberculosis (MDR-TB) calls for new treatment strategies. Biomarkers reflecting the extent of *Mycobacterium tuberculosis (Mtb)* induced pathology and normalization during anti-tuberculosis treatment (ATT) would considerably facilitate trials of new treatment regimens and the identification of patients with treatment failure^1^. The current tools for monitoring treatment of pulmonary TB are limited to clinical assessments of signs/symptoms, assessment of regular inflammatory markers (erythrocyte sedimentation rate, C-reactive protein and white blood cells), radiological changes, and repeated sampling of respiratory specimen to detect the presence of *Mtb* and their viability. The conversion of positive to negative *Mtb*-culture after 2 months treatment is the only validated predictor of the required duration of treatement and the risk of relapse^2^. Notably, this biomarker has evident limitations in children as only 30% of TB cases are culture positive at diagnosis. Recent evidence suggests that blood transcriptomes are sensitive markers of immunological changes as early as 1–2 weeks after initiation of ATT^3^,^4^,^5^. At diagnosis, TB patients are highly diverse when it comes to the manifestations and extent of disease which to some degree is reflected in smear positivity, *Mtb-*culture positivity, cavitating disease and weight, all prognostic factors of treatment response. There is a substantial body of evidence that the greater the extent of TB disease and the bacterial load in the sputum at diagnosis, the higher the risk for treatment failure or relapse^2^,^6^,^7^. Therefore, biomarkers which reflect the extent of disease are likely to have a potential to predict the ATT treatment outcome relevant to adequate individual management and prevention of multi-drug resistance. Genome-wide analysis of transcriptomes are increasingly applied in studies aiming to identify diagnostic and predictive biomarkers relevant to TB management. Surprisingly, except for *Anderson et al*. who addressed disease extent in pediatric TB according to a diagnostic algorithm that takes into account the high number of unconfirmed cases^8^, the association between transcripts that change on treatment and the extent of TB disease at diagnosis and end of treatment has not been systematically addressed^3^,^9^.

Therefore, within the setting of a randomized, double-blind, placebo-controlled longitudinal trial (rct) carried out to assess the effect of micronutrient supplementation in children with intrathoracic TB treated with ATT^10^, the present study was set up to explore 1) the association between transcriptional and translational biomarkers in WB and the extent of TB disease at diagnosis and treatment outcomes at 2 and 6 months, and 2) the potential of the biomarkers to predict treatment response at 2 and 6 months. Furthermore, we wanted to 3) explore eventual sub-clinical effects of micronutrient supplementation on immune transcripts with likely relevance in TB disease. The rationale for this was that despite clear scientific evidence that malnutrition and micronutrient deficiency are judged important risk factors for TB^11^,^12^, our original intervention trial^10^ and similar studies in other populations^13^, indicated that micronutrient supplementation does not seem to improve the clinical outcome of ATT significantly^10^. The reasons for this are unclear and may be due to the fact that micronutrient deficiency associated with malnutrition is only one factor, and may be correlated with, rather than causal for, the elevated risk. Unlike similar studies adressing the effect of micronutrient supplementation in adults^14^,^15^,^16^,^17^,^18^,^19^ and children^20^ treated for TB, sample collection in our original intervention trial^10^ allowed for detailed biomarker analyses.

## Results

### Demographic and clinical characteristics

**Figure 1:**
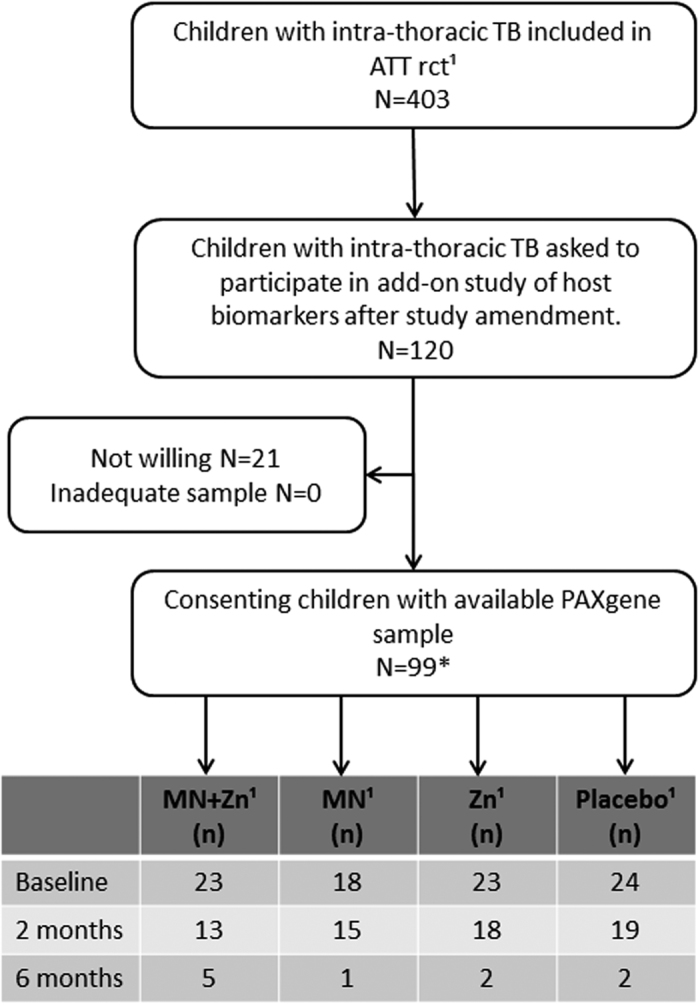
Flowchart of sample selection from ¹the randomized-controlled trial (rct) of the effect of different micronutrient (MN) supplementation on top of anti-tuberculosis therapy (ATT). As part of the ATT rct, included children were assigned to 4 intervention groups; micronutrient supplementation (MN) with or without zinc (Zn), Zn alone or placebo, and followed for 6 months^10^. Samples available for transcriptional biomarker studies from each intervention-group at baseline, and after 2 and 6 months of treatment are depicted in the table. *The sampling for the present biomarker study were possible after approval of a study amendment during the on-going inclusion to the mother study. Participation in the present add-on study required an additional consent at each study visit for additional biomarker study-specific blood-draws. This explains why 88 and not all of the 99 children have a baseline sample, and the successive loss of available samples for transcriptional biomarker studies. Altogether, 163 samples were available. The distribution of samples at baseline, and after 2 and 6 months of treatment from each intervention group, are depicted in the table for clarity.

Of the 99 children consenting to this cross-sectional study nested within a micronutrient intervention trial^10^ (Fig. 1), 49 had intrathoracic TB primarily as a result of progressive primary disease and 17 had uncomplicated lymphadenopathy. Cavities were described in 10 patients, whereas one child had miliary TB. Forty one children had *Mtb* verified by culture. Overall, the presentation and localization of TB disease in the children reflected the age distribution in the cohort; 83 children being >5 years^21^. Detailed description of the clinical characteristics are given in Table 1. There were no differences in demographic or clinical characteristics between the four micronutrient treatment groups at baseline, but a difference in the proportion of QFT positive children was found (micronutrient: 87%; micronutrient +Zinc: 50%; Zinc: 91.3%; placebo: 66.7%, p = 0.011).

### Effect of micronutrient supplements on immune biomarkers relevant in TB

**Figure 2:**
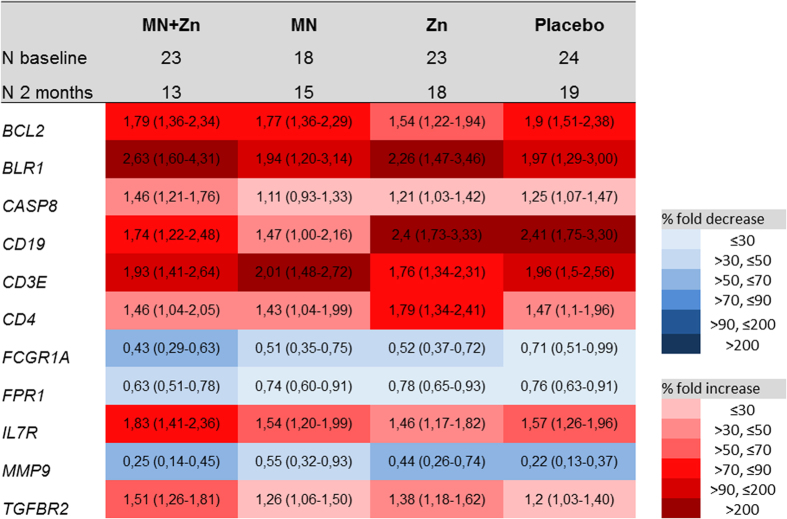
Eleven transcripts were consistently down-regulated (blue) or up-regulated (red) across all study arms. The numbers represents the mean fold changes in relative gene expression levels from baseline to 2 months of treatment, also visualized by color codes. 95% confidence intervals are given in parentheses.

As large differences in immune biomarker levels between the intervention groups would complicate the interpretation of ATT related changes, we started out by assessing changes in host transcriptional and proteomic biomarkers over time and differences between the treatment groups after 2 months of treatment (Fig. 2, Supplementary Table 1A). Although no specific biomarker pattern was found to be associated with micronutrient or zinc supplementation, 11 transcriptional biomarkers changed consistently in all groups: 3 genes were down-regulated (*FCGR1A, FPR1* and *MMP9)* and 8 genes were up-regulated (*BCL, BLR1, CASP8, CD3E, CD4, CD19, IL7R* and *TGFBR2)* (Fig. 2). With regard to the soluble cytokine biomarkers tested in stimulated WB, no significant changes were found across the groups (Supplementary Table 1B,C). This suggests the changes observed were driven by ATT rather than the trial-specific micronutrient interventions, justifying further exploration of the association between immune biomarkers and the extent of TB disease at diagnosis and treatment outcomes in the whole cohort.

### Are biomarkers that change consistently over the course of treatment associated with the extent of TB disease?

We then investigated the association between the baseline levels of the 11 transcriptional biomarkers associated with the parameters of disease extent (smear and culture positivity, cavitating disease and weight). All the 11 transcripts correlated to at least one of the modalities for disease extent. The levels of the biomarkers *FCGR1A, FPR1* and *MMP9,* correlated positively with disease extent at baseline and declined on treatment. Correspondingly, biomarkers that correlated negatively with disease extent at baseline (*BCL, BLR1, CASP8, CD3E, CD4, CD19, IL7R* and *TGFBR2)* increased on treatment, which might suggest that these markers are indicative of reconstituted immunity, or decreased pathology. Interestingly, *BLR, CD3E,* and *IL7R* associated with all the investigated parameters for disease extent (Table 2). No association with disease extent was found for the level of protein expression of IFNγ and IL-2, in response to stimulation with *Mtb*-specific antigens. Adjusting for age did not significantly influence on these associations except for *MMP9* which lost the association with *Mtb* culture positivity and cavitating disease.

### Do biomarkers that reflect the extent of TB disease associate with treatment outcomes?

In most TB patients, there is a gradual resolution of TB-related pathology on ATT. With evidence of clear association between 11 transcriptional biomarkers and the extent of TB disease in our cohort, we investigated the association of the biomarkers measured at baseline, after 2 months as well as the change from baseline to 2 months, with previously defined treatment outcomes at 2 and 6 months. Out of the 11 tested transcripts, only *BLR1* and *FCGR1A* had a significant association (Table 2): At baseline, every log unit increase in baseline *BLR1-*levels decreased the likelihood for an unfavourable outcome at 2 months by 30% (OR_*BLR1*_ 0.70, 95%CI[0.50;0.98]), but at the same time, an increase in *BLR1-*levels from baseline to 2 months increased the likelihood for an unfavourable outcome with borderline significance (OR_*BLR1*_ 1.77, 95%CI[1.00;3.13]), which likely reflects a steeper increase in BLR1 levels on treatment in children with low levels at baseline - possibly indicative of severe disease. For every log unit increase in *FCGR1A*-levels at 2 months the likelihood for an unfavourable treatment outcome at 6 months doubled (OR_*FCGR1A*_ 2.11, 95%CI[1.03;4.31]).

Adjusting for age at baseline did not influence on these associations. The distribution of treatment outcomes at 2 and 6 months in the study subjects are listed in Table 3.

### Do biomarkers that reflect the extent of TB disease predict treatment outcomes?

We therefore examined the predictive capacity of the 11 transcriptional biomarkers measured at baseline, after 2 months as well as the change from baseline to 2 months, for treatment outcomes at 2 and 6 months (Table 4). The cross-validation estimate for accuracy was best for baseline biomarker measurements and >70% for all genes except *CD4* (68%). *BCL2, CASP8, IL7R* and *TGFBR2* each had a predictive capacity of ≥75%. Both *BLR1* and *FCGR1A* associated with treatment outcomes and together their predictive capacity was 71.4%. The predictive capacity was not further increased by entering all the 11 biomarkers in the model. Likewise, testing predictive capability for treatment outcomes at 2 and 6 months by jointly entering all transcriptional biomarkers with detectable levels (20 out of 49, excluding the house-keeping gene *GAPDH*) into the model without making any a priori assumptions, did not yield any additional significant correlations. Adjusting for age at baseline slightly reduced the predictive capacity.

## Discussion

In this cohort of 99 Indian children treated for intra-thoracic TB, we report on 11 transcriptional immune biomarkers which changed consistently through the intensive phase of ATT. The three genes for which expression was down-regulated during treatment (*FCGR1A, FPR1* and *MMP9)* exhibited a positive correlation with the extent of TB disease indicating that higher levels of these transcripts probably reflect the extent of TB-induced pathology, and may be indicative of bacterial load. The 8 genes that were up-regulated during treatment (*BCL, BLR1, CASP8, CD3E, CD4, CD19, IL7R* and *TGFBR2)* exhibited a negative correlation with the extent of TB disease, strongly suggesting that lower levels of transcripts reflect a greater degree of TB-induced pathology. Whereas only *BLR1* and *FCGR1A* expression levels were associated with treatment outcomes at both 2 and 6 months, all 11 biomarkers measured at baseline showed an individual capacity exceeding 70% to predict the ATT treatment outcome at 6 months. The predictive capacity of single genes did not increase when entering all 11 markers into the leave-10-out cross validation model, suggesting that their level of expression may be driven by similar events.

This study was nested within a larger study which explored the effect of different micronutrient supplementations on biomarkers of TB host immunity. However, we were unable to identify any specific effects – instead the changes in expression detected were overwhelmingly linked to the initiation of, and response to, ATT. It is unclear whether this failure to detect a signal is because any effects from micronutrient supplementation were below the threshold of detection by this study approach, or due to the biomarkers selected being sub-optimal for detecting the effect of micronutrients on host immune responses, or to limited sample size.

Previous studies have explored changes in transcriptional biomarkers on ATT in adults^2^,^3^,^4^,^5^,^22^,^23^,^24^,^25^ but studies in children are limited^5^,^26^. Research for predictive markers of treatment response is driven by the few tools available to guide decision-making during the prolonged course of ATT^2^. Notably, the only generally-accepted marker for treatment failure currently is lack of culture conversion at 2 months^27^. However, in addition to requiring weeks to get a final negative culture result, this parameter is of no use in the 70% pediatric TB cases that are culture negative at diagnosis^28^, thus leaving us with no tools to assess the early response to ATT in children. It is possible that specific host immune factors present during these first 2 months or even before the initiation of treatment, may help predicting treatment outcomes. Identification of such objectively-measured factors could pave the way for tailored individual treatment as well as facilitate the evaluation of the ongoing and planned studies of new treatment regimens in children^29^. The present study is the first to directly assess how a panel of biomarkers associate with established parameters of TB disease extent and treatment outcomes in children. The reported consistencies between biomarkers and the extent of disease provide evidence for the assumption that the measured transcripts are biomarkers which truly reflect ongoing *Mtb*-related pathology. Treatment success is the key in limiting the emerging epidemic of multi-drug resistant TB, and depends on close monitoring of possible treatment failure or relapse. Biomarkers with a capacity to predict treatment response could enable more targeted use of limited health care resources to persons at risk. The impact on clinical management would be greatest for biomarkers with a predictive potential when measured at the initiation of treatment^22^. In the present study, ten out of 11 transcriptional biomarkers predicted the treatment outcome at 6 months with an accuracy above 70%. *CASP8, FPR1, IL7R* and *TGFRB2* each had a predictive accuracy of ≥75% in a leave-10-out cross validation. Notably, baseline levels and changes in expression of *BLR1* and *FCGR1* were associated with treatment outcomes after 6 months, when assessed by logistic regression.

We report a positive correlation between the *FCGR1A*-levels at baseline and the extent of TB disease when assessed by smear or *Mtb*-culture positivity, or caviting disease. Primate models suggest that the extent of TB disease is closely correlated with bacterial load^30^. Furthermore, *FCGR1A* decreased with treatment, but for every log unit of decrease in *FCGR1A* at 2 months we observed a doubled likelihood for an unfavourable outcome at 6 months. *FCGR1A* is an interferon-inducible gene highly expressed by myeloid cells like macrophages and neutrophils. Our findings are consistent with increasingly strong evidence of an up-regulation of interferon-inducible genes in TB patients^4^,^8^,^24^,^31^,^32^,^33^ and decline during treatment. A decline in *FCGR1A*-levels following treatment initiation has also been reported by others^9^. Notably, *FCGR1A* has also been shown to have a diagnostic potential for TB disease in various populations^32^,^34^,^35^, including children^25^,^36^ and is robust even in HIV co-infected subjects^32^,^34^. Very recently, *FCGR1A* was included in a 16-gene signature that predicted TB progression in latently infected individuals with a sensitivity of 71.2% within the 6–12 months preceding active TB disease^25^. Indeed, markers with a classifying potential in both diagnosis and prediction of treatment outcome are highly interesting for future point-of-care (POC) tools in order to facilitate both diagnosis and real-time evaluation of treatment response.

Interestingly, some of the biomarkers which associated with the extent of TB disease that changed consistently with treatment in the present study, are typically thought of as markers of B-cell mediated immunity: *BLR1, CD19* and *FCGR1A.* (Supplementary Table 1). Although the *FCGR1A* gene encodes the receptor which mediates the effect of binding of the Fc-region of Ig-G on B-cells, the contribution to the *FCGR1A* level in peripheral blood in the setting of TB is probably mostly due to a high expression on myeloid cells^24^ as previously discussed. B-cells were not given much attention in TB pathogenesis until Ulrichs and Kaufmann described the emergence of tertiary lymphoid structures in the vicinity of lung granulomas of TB patients^37^. The interest increased when Berry *et al*.^24^. revealed decreased abundance of B-cell transcripts in the RNA signature of TB patients, which has later been supported by others^33^,^38^,^39^. A recent publication also found an increase in the expression of B-cell markers from 4 to 26 weeks of ATT^3^.

Of the other 11 transcripts that consistently changed with treatment, an increase in *CD19* expression on treatment has also been described by others^3^,^9^. Interestingly, of the markers of disease extent investigated here, *CD19* only exhibited a small positive correlation with the BMIZ-score.

Unbiased RNA sequencing may offer some advantage compared with the dcRT-MLPA method. The dcRT-MLPA method was however, chosen for its suitability for low-cost hypothesis-testing of biomarkers identified as a result of joint efforts in TB biomarker discovery by the partners in Bill and Melinda Gates foundation (GC6). The genes previously identified, have shown differential expression between TB patients, and healthy subjects with or without latent *Mtb* infection (LTBI), either in studies based on unbiased approaches or biased approaches where the markers were selected for their known/suggested role in TB pathogenesis (Supplementary Table 2). Moreover, the low volume of sample material required for the dcRT-MLPA was also an important consideration important given the limited amount of sample drawn per child per time-point^9^,^40^. As we did not apply any multiplicity adjustment of p-values there is an increased risk of reporting false positive findings, but this approach was deliberately chosen to reduce the risk that we might overlook any potential associations. Although age adjustments slightly reduced the predictor capacity of the single transcripts and signatures tested, it had minor effect on the associations with TB disease extent and treatment outcome. This is indeed encouraging as immunological diagnostics are well known to be less reliable in young children^41^, and the fact that, diagnostic or monitoring tools requiring age adjustments complicates future clinical application. There is also a possibility that the reported changes, the correlations with the extent of TB disease and the predictive capacities of the identified transcripts discovered here are cohort-specific. Because of these limitations, the results need to be further evaluated and confirmed. Nonetheless, the consistent evidence for *FCGR1A* as a biomarker of the extent of TB disease is consistent across many studies, especially as previous studies have been done mostly in African cohorts of adolescents and adults, whereas the present study adds valuable evidence of relevance in Indian children.

Whereas, previous studies have explored the changes in transcriptional biomarkers on ATT, the present study is the first to directly assess how the biomarkers associate with established parameters of TB disease extent and treatment outcomes in children. Measured directly *ex-vivo* by a robust, accurate and cheap high-throughput technique, we found that low levels of *BLR1* at baseline were indicative of more severe TB disease and increased the likelihood of an unfavourable treatment outcome at 2 months. High levels of *FCGR1A* measured after 2 months increased the likelihood of an unfavourable treatment outcome at 6 months. Together, these markers could serve as monitoring and predictive markers of treatment response to optimize the individual treatment and minimize the risk of relapse.

## Materials and Methods

### Source population

This study was cross-sectional and nested within a randomized, double-blind prospective controlled trial conducted from January 2008 to June 2012 at the All India Institute of Medical Sciences and Kalawati Saran Children Hospital associated with Lady Hardinge Medical College in Delhi, India for which the study details are described elsewhere^10^. Briefly, children aged 6 months to 15 years who presented with any of: cough and/or fever >2 weeks with no improvement after a 7–10 day course of amoxicillin; recent unexplained weight loss/failure to thrive, fatigue/lethargy (reduced playfulness), or clinical symptoms and a history of close contact with an adult patient with TB, were screened for TB at admittance to the pediatric ward. Children, critically ill at admission, with significant comorbidity (including HIV), a history of anti-tuberculosis treatment (ATT), and/or contact with a TB case with known drug resistance were excluded. In total, 403 children with a diagnosis of intra-thoracic TB were included and randomly assigned to one of four groups; micronutrient supplementation (MN) with or without zinc (Zn), zinc only or a placebo, initiated at the same time as ATT. Twenty-two children were lost to follow-up (5.5%), leaving 381 (94.5%) children who completed the 6-month follow-up.

### Diagnostic assessment and follow-up

Medical history (including BCG vaccination status, history of TB and/or TB exposure), clinical, demographic, and anthropometric data were recorded. A TST was performed by a trained nurse (5 TU/0.1 mL tuberculin; Span Diagnostics, Surat, India) and read after 48–72 hours; an induration ≥10 mm was defined as positive. Peripheral blood (3 ml) was drawn for the QuantiFERON^®^-TB Gold In-Tube (QFT) test (Cellestis, Australia), according to the manufacturer’s instructions. A CXR, anterioposterior and lateral views, were recorded and interpreted by three independent radiologists. Agreement by at least two radiologists was required for the diagnostic inclusion criteria of intra-thoracic TB (findings of hilar or mediastinal adenopathy, consolidation, cavitation, miliary shadows and/or pleural effusion; criteria similar to the diagnostic recommendation by the revised National Tuberculosis Control Progam of India (NTP))^42^. From suspected TB cases, gastric aspirates (GA) and induced sputa (IS) were collected on two consecutive days for fluorescent microscopy (Auramine) and culture (Mycobacterial Growth Indicator Tube, BD) and processed after decontamination, as described previously^43^. The prevalence of HIV infection was assessed anonymously as part of the main study (TB cases only) after pretest counselling as per national guidelines^44^ and found to be <1%. Children with HIV co-infection were excluded from the main study.

Included children were assessed every second week until 2 months of treatment and, thereafter, every 4 weeks until they completed the assigned ATT as per recommendation of the NTP (Category 1 treatment: isoniazid, rifampicin, pyrazinamide and ethambutol for 2 months followed by isoniazid and rifampicin for 4 months; Category 3 treatment: isoniazid, rifampicin and pyrazinamide for 2 months followed by 2 drugs for 4 months). Clinical and anthropometric data were recorded at every visit and CXR performed at 2 and 6 months.

### Study population

Of the 403 children with intrathoracic TB included in the main study, the last 120 included were asked to participate in the present study, which was made possible after obtaining additional funding and a protocol amendment. Consent to collect an additional blood draw (PAXgene Blood RNA Tubes; PreAnalytiX, Hilden, Germany) was given by parent/guardian for 99 of the children for one or more of the time points. Longitudinal samples (163 altogether) were included in the present study and assessed for transcriptional and translational biomarkers. Figure 1 shows the flowchart of availability of samples at each timepoint for each of the 4 treatment arms.

### Definitions of extent of TB disease and treatment outcomes

The extent of TB disease was evaluated at baseline (before the initiation of treatment) and treatment outcomes were evaluated after 2 and 6 months, applying established parameters known to be linked with the risk of relapse or treatment failure, namely smear positivity (in any of 2 IS or 2 GA samples), *Mtb*-culture positivity, cavitating disease and weight^2^,^6^,^7^. A favourable 2 month outcome was defined as *Mtb*-culture negativity, at least some improvement on CXR and weight gain whereas an unfavourable outcome was having either *Mtb*-culture positivity, worsening or no improvement on CXR, or no weight gain. The outcomes at 6 months were defined similarly – the only difference being CXR for which a significant clearance was required for a favourable outcome. Weight change was assessed by the standardized WHO Body Mass Index-for-age Z-score (BMIZ), which was chosen over weight-for-age Z-score because it contains height and therefore provides more accurate information^45^. The change in radiological lesions was judged by two independent radiologists and a third radiologist was included in cases with disagreement.

### RNA extraction

Total RNA was extracted from the PAXgene blood collection tubes using the ‘PAXgene Blood RNA kit’ with RNase free DNase on-column digestion (PreAnalytiX, Hilden, Germany) according to the manufacturer’s instructions. The total RNA concentration and purity (A_260/280_nm ratio) were measured using a Nanodrop spectrophotometer (Thermoscientific, Wilmington, Delaware, USA) and ranged between 0.2–13.7 μg (average 2.1 ± 0.64 μg), and supplemented by agarose gel electrophoresis.

### Dual colour Reverse-Transcriptase Multiplex Ligation-dependent Probe Amplification (dcRT-MLPA)

As available blood samples were limited, we used a novel high-throughput technique, which requires 130–150 ng of total RNA for a predefined panel of genes. The dcRT-MLPA method can rapidly profile multiple host genes with a dynamic range and sensitivity comparable to real-time qPCR and RNA sequencing at a cost of 5–7 euros per sample^40^. The genes included in the dcRT-MLPA were pre-selected based on joint efforts in TB biomarker discovery by the partners in Bill and Melinda Gates foundation GC6 (Supplementary Table 2)^9^. The protocol has previously been described in detail^9^ and therefore is only briefly discussed here. A table of the 49 immune-related genes analyzed for expression and their suspected relationship to TB pathogenesis have previously been published^36^ (Supplementary Table 2). Probes and primers were obtained from the Department of Infectious Diseases, Leiden University Medical Center, Leiden, The Netherlands. Primers and probes were synthesized by Sigma-Aldrich Chemie (Zwijndrecht, the Netherlands) and MLPA SALSA reagents were from MRC-Holland (Amsterdam, the Netherlands). Samples with a concentration <50 ng/μl were concentrated at 45 °C using a speed-vacuum concentrator (Eppendorf AG, Hamburg, Germany). A positive control (using synthetic oligonucleotides as hybridization templates) and a commercial Human Universal Reference RNA were included on each plate. All samples (n = 163) were run in duplicate. The amplified PCR products were diluted 1:10 with nuclease-free H_2_O and added to a mixture of Hi-Di-Formamide with 400HD ROX size standard. The PCR fragments were denatured at 95 °C for 5 min, cooled on ice and analyzed on a 3730 capillary sequencer (Life Technologies, Carlsbad, California, USA). Data were analyzed using GeneMapper version 4.0 (Life Technologies, Carlsbad, California, USA) with adjustments of the default peak detection settings if required. The peak areas of replicates were averaged, normalized against *GAPDH* and log2 transformed as described^46^.

### Multiplex bead array (bioplex)

Biomarkers at the translational level (IFNα2, IFNγ, IL1β, IL2, IL4, IL5, IL6, IL7, IL8, IL10, IL12p40, IL12p70, IL13, IL17A, IP10, MCP1, MCP3 and TNFα) were analyzed in peripheral WB stimulated with MTB-specific antigens: Early Secretory Antigenic Target-6 (ESAT-6), Culture Filtrate Protein-10 (CFP10) and TB antigen 7.7 (QFT supernatants) and analyzed using the ‘Human cytokines 18-plex’ kit (Milliplex MAP Human Cytokine/Chemokine kit, Merck Millipore, Missouri, USA) according to the manufacturer’s instructions.

### Statistical analysis

Differences in clinical characteristics between the intervention groups at baseline were assessed by Pearson’s chi-square test or Fisher’s exact test, where appropriate.

Treatment outcome variables were treated as binary outcomes. BMIZ was evaluated both as a binary variable (dichotomized around the cut-off <−2 which marks underweight)^45^ and as a continuous variable.

We compared the longitudinal profiles between intervention groups after 2 and 6 months treatment by means of linear mixed models, which are ideally suited for analysis of repeated measurements with missing values. Changes in immune biomarkers over time within intervention groups were also assessed by the same models. In addition to the intervention-time interaction with a common baseline level, these models included adjustments for age and gender as fixed effects and for subject-specific differences as random effects. Measurements that fell below the lower detection limit or above the upper detection limit were accommodated in models as left- and right-censored observations, respectively^47^. Models were fitted to base 2 logarithm(log2)-transformed measurements. Differences in mean levels were evaluated based on posthoc t-tests derived from the linear mixed models and reported as back-transformed fold changes.

For the biomarkers, which consistently changed with ATT, the association between their baseline levels and the extent of TB disease was assessed by logistic regression and Spearman’s Rank Correlation where appropriate. Furthermore, values at baseline, at 2 months, and the corresponding change scores were all analyzed while adjusting for micronutrient supplementation by 1) separate logistic regression models to evaluate the possible association with the treatment outcomes at 2 and 6 months, 2) by a leave-10-out cross-validation to evaluate their capacity to predict the treatment outcomes at 2 and 6 months, and finally by jointly entering all transcriptional biomarkers with detectable levels (20 out of 49, excluding the house-keeping gene *GAPDH*) into the model without making any a priori assumptions. All analyses were performed with and without adjustment for age at baseline.

The statistical environment R (R Core Team, 2014) and IBM SPSS version 21 (IBM, Bergen, Norway) were used for the analyses. Dot plots were created using GraphPad Prism 5 (GraphPad Software, Inc. La Jolla, California, USA). P-values <0.05 were considered significant.

### Ethics statement

The study protocol was approved by the respective institutional ethics committees at the All India Institute of Medical Sciences, New Delhi and Kalawati Saran hospital, a children hospital associated with Lady Hardinge Medical College in New Delhi, India. The main randomised control trial in which this biomarker study is nested, was registered at clinicaltrials.gov (NCT00801606). Written informed consent was obtained from parents/guardians as well as written assent for participants >7 years. All methods were performed in accordance with the relevant guidelines and regulations.

## Additional Information

**How to cite this article**: Jenum, S. *et al*. BLR1 and FCGR1A transcripts in peripheral blood associate with the extent of intrathoracic tuberculosis in children and predict treatment outcome. *Sci. Rep.* **6**, 38841; doi: 10.1038/srep38841 (2016).

**Publisher's note:** Springer Nature remains neutral with regard to jurisdictional claims in published maps and institutional affiliations.

## Supplementary Material

Supplementary Tables

## Figures and Tables

**Table 1:** Demographic and clinical baseline characteristics of 99 children with intra-thoracic TB and samples analyzed for transcriptional biomarkers (dcRT-MLPA).

Baseline characteristics		Distribution
Sociodemographics and medical history		
Gender	*Male (n)*	45
Age	*mean age in months (St.dev)*	107,8 (46,0)
	*≤5 years (n)*	16
	*>5 to ≤10 years (n)*	40
	*>10 to 16 years (n)*	43
Known contact with TB patient *(n)*		35
BCG vaccination		
	*Yes (n)*	82
	*No (n)*	16
	*Not known (n)*	1
Symptoms		
Cough ≥2 weeks (n)		63
Fever (n)		77
Weight loss (n)		74
Loss of appetite (n)		74
Anthropometric data		
Body Mass Index Z-score, mean (St.dev)		−2,28 (1,46)
Weight-for-age Z-score, mean (St.dev)		−2,54 (1,68)
Diagnosis and treatment		
Smear positivityˇ (n)		13
*Mtb-*culture positive° (n)		41
NTM culture positive^a^ (n)		7
Finding on Chest X-ray		
	*uncomplicated LN disease (n)*	17
	*LN disease with collapse (n)*	2
	*progressive primary disease (n)*	49
	*primary pulmonary complex (n)*	11
	*miliary TB (n)*	1
	*pleural effusion (n)*	8
	*cavitation disease (n)*	10
	*calcification (n)*	1
Lymph node swelling (n)		19
Tuberculin Skin Test		
	*mean in mm (St.dev)*	15,1 (8,1)
	*≥10 mm (n)*	96
QuantiFERON Gold In-tube		
	*Positive (n)*	76
	*Negative (n)*	22
	*Indeterminate (n)*	1
Anti-tuberculosis treatment		
	*Category 1^b^ (n)*	75
	*Category 3^c^ (n)*	24
Micronutrient supplement		
	*Micronutrients and Zinc (n)*	25
	*Micronutrients (n)*	21
	*Zinc (n)*	26
	*Placebo (n)*	27

Of 2 induced sputum samples and 2 gastric aspirate samples taken on 2 consecutive days at least ˇone specimen positive for acid-fast bacilli, °one culture samples with growth of *Mycobacterium tuberculosis or*^a^Non-tuberculous mycobacteria. ^b^2 months with isoniazid, rifampicin, pyrazinamide and etambuthol followed by 4 months of isoniazid, rifampicin. ^c^2 months with isoniazid, rifampicin and pyrazinamide followed by 4 months of 2 drugs.

**Table 2:** Associations between baseline measurements of each of the 11 biomarkers (BM) that consitently changed with ATT and the extent of TB disease at the time of diagnosis (before the initiation of treatment).

BM change with treatment	BM entered in the model	Smear positivityˇ		*Mtb* culture positive°		Cavitating disease		Body Mass Index Z-score <−2		Body Mass Index Z-score, continous scale		Association with treatment outcomes	
		**OR**	**95% CI for OR**	**OR**	**95% CI for OR**	**OR**	**95% CI for OR**	**OR**	**95% CI for OR**	**Rho**	**p**	**2 months**	**6 months**
Decrease	*FCGR1A*	ns		3,14	(1,584–6,21)	ns		ns		ns		YES^a^	YES^a^
	*FPR1*	9,78	(2,09–45,8)	ns		ns		2,83	(1,13–7,14)	ns		ns	ns
	*MMP9*	ns		1,44	(1,02–2,03)	1,82	(1,06–3,12)	ns		–0,24	0,024	ns	ns
Increase	*BCL2*	0,3	(0,1–0,92)	ns		0,12	(0,03–0,49)	ns		ns		ns	ns
	*BLR1*	0,47	(0,32–0,70)	0,46	(0,28–0,74)	0,5	(0,33–0,74)	ns		0,24	0,026	YES^b^	ns
	*CASP8*	ns		ns		0,13	(0,03–0,68)	ns		ns		ns	ns
	*CD3E*	0,42	(0,23–0,78)	0,55	(0,33–0,92)	0,38	(0,20–0,75)	ns		0,23	0,037	ns	ns
	*CD4*	0,37	(0,18–0,78)	ns		0,45	(0,23–0,88)	ns		ns		ns	ns
	*CD19*	ns		ns		ns		ns		0,22	0,043	ns	ns
	*IL7R*	0,25	(0,11–0,56)	0,38	(0,21–0,69)	0,22	(0,09–0,53)	ns		0,22	0,04	ns	ns
	*TGFBR2*	0,22	(0,05–0,94)	0,11	(0,03–0,37)	0,02	(0,003–0,19)	ns		ns		ns	ns

Of 2 induced sputum samples and 2 gastric aspirate samples taken on 2 consecutive days at least ˇone specimen positive for acid-fast bacilli, °one culture samples with growth of Mycobacterium tuberculosis. ^a^2-month *FCGR1A*-value; positive association, details given in the text. ^b^Baseline *BLR1*-value; negative association, and change from BL to 2 months; positive association, details given in the text.

Unadjusted odds ratio estimates and 95% confidence intervals is given for categorical variables and Spearman’s Rho and corresponding p-value is given for continous variables. ns signifies non-significant associations.

**Table 3:** The distribution of treatment outcomes after 2 and 6 months of antituberculosis therapy (ATT) in the 99 included children with intrathoracic TB.

Variables of treatment response	2 monthsN = 99	6 monthsN = 99
Smear positivityˇ (n)	5	1
not done	57	74
*missing*	*0*	*1*
MTB culture positive° (n)	16	7
*missing*	*0*	*2*
CXR		
significant clearance	37	74
some improvement	37	19
no improvement	23	2
worsening	2	0
*missing*	*0*	*4*
CXR recoded^a^		
unfavourable	62	21
*missing*	*0*	*4*
Change in Body Mass Index Z-score		
>0 (weight gain)	80	90
≤0 (no weight gain/weight loss)	16	9
*missing*	*3*	*9*
Change in weight-for-age Z-score		
>0 (weight gain)	81	91
≤0 (no weight gain/weight loss)	18	8
*missing*	*0*	*0*
Extension of ITP at 2 months	16	na
*missing*	*0*	*0*
Any symptom^b^	24	na
*missing*	*0*	*0*

Of 2 induced sputum samples and 2 gastric aspirate samples taken on 2 consecutive days at least ˇone specimen positive for acid-fast bacilli, °one culture samples with growth of *Mycobacterium tuberculosis.*^a^An unfavourable 2 month outcome was having either of MTB culture positivity, no improvement/worsening at CXR or no weight gain. An unfavourable 6 month outcome was having either of MTB culture positivity, lack of significant improvement at CXR or no weight gain. ^b^Any of the parent-reported symptoms cough, fever, loss of appetite, weight loss, lymphnode swelling at 2 months.

**Table 4:** The capacity to predict 2 and 6 months treatment outcomes by 11 biomarkers (BM) which changed consistently on ATT was analyzed applying logistic regression, leave-10-out cross-validation while adjusting for micronutrient supplementation.

BM entered in the model		Prediction by BM measured at baseline				Prediction by BM measured after 2 months		Prediction by the change in BM from baseline to 2 months	
		**Outcome at 2 months**		**Outcome at 6 months**		**Outcome at 6 months**		**Outcome at 6 months**	
		**Int. est.**^a^	**Cross-validation**	**Int. est.**^a^	**Cross-validation**	**Int. est.**^a^	**Cross-validation**	**Int. est.**^a^	**Cross-validation**
single biomarkers	*BCL2*	0,614	0,568	0,762	**0,75**	0,651	0,587	0,759	0,648
	*BLR1*	0,636	0,614	0,738	**0,702**	0,619	0,587	0,704	0,63
	*CASP8*	0,614	0,58	0,75	**0,762**	0,635	0,619	0,704	0,685
	*CD3E*	0,636	0,568	0,726	**0,714**	0,651	0,587	0,741	0,667
	*CD4*	0,602	0,545	0,714	0,679	0,603	0,556	0,685	0,63
	*CD19*	0,625	0,557	0,762	**0,714**	0,651	0,587	0,667	0,667
	*FCGR1A*	0,625	0,591	0,738	**0,702**	0,651	0,635	0,685	0,537
	*FPR1*	0,625	0,58	0,75	**0,714**	0,651	0,603	0,722	0,667
	*IL7R*	0,625	0,557	0,75	**0,75**	0,651	0,587	0,741	0,667
	*TGFBR2*	0,614	0,591	0,75	**0,75**	0,651	0,571	0,704	0,574
	*MMP9*	0,58	0,557	0,738	**0,702**	0,698	0,683	0,778	**0,759**
signatures	*FCGR1A & BLR1*	0,648	0,568	0,738	**0,714**	0,667	0,603	0,685	0,537
	11 markers*	0,727	0,523	0,762	0,679	0,73	0,619	0,759	0,593
	10 markers**	0,705	0,523	0,786	0,643	0,746	0,651	0,759	0,593

^a^Internal estimate of accuracy. ^*^Cross-validation of the combination of all 11 biomarkers that consistently changed with treatment (BCL2, BLR1, CASP8, CD19, CD3E, CD4, FCGR1A, FPR1, IL7R, MMP9, and TGFBR2). ^**^Cross-validation of the combination of the same markers as above, but excluding CD4.

Biomarkers measured at the start of ATT (baseline), at 2 months, and the corresponding change in score were assessed. Prediction accuracy in the cross-validation estimates are bolded.

## References

[b1] Wallis R. S. . Tuberculosis–advances in development of new drugs, treatment regimens, host-directed therapies, and biomarkers. The Lancet. Infectious diseases 16, e34–46, doi: 10.1016/s1473-3099(16)00070-0 (2016). 27036358

[b2] Wallis R. S. . Tuberculosis biomarkers discovery: developments, needs, and challenges. Lancet Infect. Dis 13, 362–372 (2013). 23531389 10.1016/S1473-3099(13)70034-3

[b3] Cliff J. M. . Distinct phases of blood gene expression pattern through tuberculosis treatment reflect modulation of the humoral immune response. The Journal of infectious diseases 207, 18–29, doi: 10.1093/infdis/jis499 (2013). 22872737

[b4] Bloom C. I. . Detectable changes in the blood transcriptome are present after two weeks of antituberculosis therapy. PLoS One 7, e46191, doi: 10.1371/journal.pone.0046191 (2012). 23056259 PMC3462772

[b5] Sweeney T. E., Braviak L., Tato C. M. & Khatri P. Genome-wide expression for diagnosis of pulmonary tuberculosis: a multicohort analysis. The Lancet. Respiratory medicine 4, 213–224, doi: 10.1016/s2213-2600(16)00048-5 (2016). 26907218 PMC4838193

[b6] Benator D. . Rifapentine and isoniazid once a week versus rifampicin and isoniazid twice a week for treatment of drug-susceptible pulmonary tuberculosis in HIV-negative patients: a randomised clinical trial. Lancet 360, 528–534 (2002). 12241657 10.1016/s0140-6736(02)09742-8

[b7] Hesseling A. C. . Baseline sputum time to detection predicts month two culture conversion and relapse in non-HIV-infected patients. International Journal of Tuberculosis & Lung Disease 14, 560–570 (2010). 20392348

[b8] Anderson S. T. . Diagnosis of childhood tuberculosis and host RNA expression in Africa. The New England journal of medicine 370, 1712–1723, doi: 10.1056/NEJMoa1303657 (2014). 24785206 PMC4069985

[b9] Joosten S. A. . Identification of biomarkers for tuberculosis disease using a novel dual-color RT-MLPA assay. Genes Immun 13, 71–82 (2012). 21956656 10.1038/gene.2011.64

[b10] Lodha R. . Effect of micronutrient supplementation on treatment outcomes in children with intrathoracic tuberculosis: a randomized controlled trial. Am J Clin Nutr 100, 1287–1297, doi: 10.3945/ajcn.113.082255 (2014). 25332327

[b11] Vanden Driessche K., Persson A., Marais B. J., Fink P. J. & Urdahl K. B. Immune vulnerability of infants to tuberculosis. Clinical & developmental immunology 2013, 781320, doi: 10.1155/2013/781320 (2013). 23762096 PMC3666431

[b12] Lonnroth K. & Raviglione M. Global epidemiology of tuberculosis: prospects for control. Semin. Respir. Crit Care Med 29, 481–491 (2008). 18810682 10.1055/s-0028-1085700

[b13] Sinclair D., Abba K., Grobler L. & Sudarsanam T. D. Nutritional supplements for people being treated for active tuberculosis. The Cochrane database of systematic reviews. Cd006086, doi: 10.1002/14651858.CD006086.pub3 (2011). 22071828

[b14] Salahuddin N. . Vitamin D accelerates clinical recovery from tuberculosis: results of the SUCCINCT Study [Supplementary Cholecalciferol in recovery from tuberculosis]. A randomized, placebo-controlled, clinical trial of vitamin D supplementation in patients with pulmonary tuberculosis’. BMC infectious diseases 13, 22, doi: 10.1186/1471-2334-13-22 (2013). 23331510 PMC3556334

[b15] Martineau A. R. . High-dose vitamin D(3) during intensive-phase antimicrobial treatment of pulmonary tuberculosis: a double-blind randomised controlled trial. Lancet 377, 242–250, doi: 10.1016/s0140-6736(10)61889-2 (2011). 21215445 PMC4176755

[b16] Wejse C. . Vitamin D as supplementary treatment for tuberculosis: a double-blind, randomized, placebo-controlled trial. American journal of respiratory and critical care medicine 179, 843–850, doi: 10.1164/rccm.200804-567OC (2009). 19179490

[b17] Ralph A. P. . L-arginine and vitamin D adjunctive therapies in pulmonary tuberculosis: a randomised, double-blind, placebo-controlled trial. PLoS One 8, e70032, doi: 10.1371/journal.pone.0070032 (2013). 23967066 PMC3743888

[b18] Karyadi E. . A double-blind, placebo-controlled study of vitamin A and zinc supplementation in persons with tuberculosis in Indonesia: effects on clinical response and nutritional status. The American journal of clinical nutrition 75, 720–727 (2002). 11916759 10.1093/ajcn/75.4.720

[b19] Range N. . The effect of multi-vitamin/mineral supplementation on mortality during treatment of pulmonary tuberculosis: a randomised two-by-two factorial trial in Mwanza, Tanzania. The British journal of nutrition 95, 762–770 (2006). 16571156 10.1079/bjn20051684

[b20] Mehta S. . A randomized trial of multivitamin supplementation in children with tuberculosis in Tanzania. Nutrition journal 10, 120, doi: 10.1186/1475-2891-10-120 (2011). 22039966 PMC3229564

[b21] Perez-Velez C. M. & Marais B. J. Tuberculosis in children. N. Engl. J. Med 367, 348–361, doi: 10.1056/NEJMra1008049 [doi] (2012). 22830465

[b22] Walzl G., Ronacher K., Hanekom W., Scriba T. J. & Zumla A. Immunological biomarkers of tuberculosis. Nat. Rev. Immunol 11, 343–354 (2011). 21475309 10.1038/nri2960

[b23] Whitworth H. S., Aranday-Cortes E. & Lalvani A. Biomarkers of tuberculosis: a research roadmap. Biomark. Med 7, 349–362, doi: 10.2217/bmm.13.53 [doi] (2013). 23734796

[b24] Berry M. P. . An interferon-inducible neutrophil-driven blood transcriptional signature in human tuberculosis. Nature 466, 973–977 (2010). 20725040 10.1038/nature09247 PMC3492754

[b25] Zak D. E. . A blood RNA signature for tuberculosis disease risk: a prospective cohort study. Lancet. doi: 10.1016/s0140-6736(15)01316-1 (2016). PMC5392204 27017310

[b26] Kleinsteuber K. . Decreased expression of miR-21, miR-26a, miR-29a, and miR-142-3p in CD4(+) T cells and peripheral blood from tuberculosis patients. PLoS One 8, e61609, doi: 10.1371/journal.pone.0061609 (2013). 23613882 PMC3628900

[b27] Wallis R. S. . Biomarkers for tuberculosis disease activity, cure, and relapse. Lancet Infect. Dis 9, 162–172 (2009). 19246020 10.1016/S1473-3099(09)70042-8

[b28] Dodd L. E. & Wilkinson R. J. Diagnosis of paediatric tuberculosis: the culture conundrum. Lancet Infect. Dis 13, 3–4, doi: S1473-3099(12)70290-6 [pii];10.1016/S1473-3099(12)70290-6 [doi] (2013). 23134696 10.1016/S1473-3099(12)70290-6

[b29] Seddon J. A., McKenna L., Shah T. & Kampmann B. Recent Developments and Future Opportunities in the Treatment of Tuberculosis in Children. Clinical infectious diseases: an official publication of the Infectious Diseases Society of America **61**Suppl 3, S188–199, doi: 10.1093/cid/civ582 (2015). 26409282

[b30] Lin P. L. . Sterilization of granulomas is common in active and latent tuberculosis despite within-host variability in bacterial killing. Nat. Med 20, 75–79, doi: nm.3412 [pii];10.1038/nm.3412 [doi] (2014). 24336248 10.1038/nm.3412 PMC3947310

[b31] Bloom C. I. . Transcriptional blood signatures distinguish pulmonary tuberculosis, pulmonary sarcoidosis, pneumonias and lung cancers. PLoS One 8, e70630, doi: 10.1371/journal.pone.0070630 (2013). 23940611 PMC3734176

[b32] Kaforou M. . Detection of tuberculosis in HIV-infected and -uninfected African adults using whole blood RNA expression signatures: a case-control study. PLoS Med 10, e1001538, doi: 10.1371/journal.pmed.1001538 (2013). 24167453 PMC3805485

[b33] Ottenhoff T. H. . Genome-wide expression profiling identifies type 1 interferon response pathways in active tuberculosis. PLoS One 7, e45839, doi: 10.1371/journal.pone.0045839 (2012). 23029268 PMC3448682

[b34] Sutherland J. S. . Differential gene expression of activating Fcgamma receptor classifies active tuberculosis regardless of human immunodeficiency virus status or ethnicity. Clinical microbiology and infection: the official publication of the European Society of Clinical Microbiology and Infectious Diseases 20, O230–238, doi: 10.1111/1469-0691.12383 (2014). 24205913

[b35] Mihret A. . Combination of gene expression patterns in whole blood discriminate between tuberculosis infection states. BMC infectious diseases 14, 257, doi: 10.1186/1471-2334-14-257 (2014). 24885723 PMC4041060

[b36] Jenum S. . Approaching a diagnostic point-of-care test for pediatric tuberculosis through evaluation of immune biomarkers across the clinical disease spectrum. Scientific reports 6, 18520, doi: 10.1038/srep18520 (2016). 26725873 PMC4698754

[b37] Ulrichs T. & Kaufmann S. H. New insights into the function of granulomas in human tuberculosis. The Journal of pathology 208, 261–269, doi: 10.1002/path.1906 (2006). 16362982

[b38] Ottenhoff T. H. New pathways of protective and pathological host defense to mycobacteria. Trends Microbiol 20, 419–428, doi: 10.1016/j.tim.2012.06.002 (2012). 22784857

[b39] Joosten S. A., Fletcher H. A. & Ottenhoff T. H. A Helicopter Perspective on TB Biomarkers: Pathway and Process Based Analysis of Gene Expression Data Provides New Insight into TB Pathogenesis. PLoS. One 8, e73230, doi: 10.1371/journal.pone.0073230[doi];PONE-D-13-23753 [pii] (2013). 24066041 PMC3774688

[b40] Haks M. C., Goeman J. J., Magis-Escurra C. & Ottenhoff T. H. Focused human gene expression profiling using dual-color reverse transcriptase multiplex ligation-dependent probe amplification. Vaccine 33, 5282–5288, doi: 10.1016/j.vaccine.2015.04.054 (2015). 25917681

[b41] Pai M. . Gamma interferon release assays for detection of Mycobacterium tuberculosis infection. Clin. Microbiol. Rev 27, 3–20, doi: 27/1/3 [pii];10.1128/CMR.00034-13 [doi] (2014). 24396134 10.1128/CMR.00034-13 PMC3910908

[b42] Chauhan L. S., Arora V. K., Central Tb Division D. G. o. H. S. M. o. H., Family W. & Indian Academy of P. Management of pediatric tuberculosis under the Revised National Tuberculosis Control Program (RNTCP). Indian pediatrics 41, 901–905 (2004). 15475631

[b43] Mukherjee A. . Ambulatory gastric lavages provide better yields of Mycobacterium tuberculosis than induced sputum in children with intrathoracic tuberculosis. The Pediatric infectious disease journal 32, 1313–1317, doi: 10.1097/INF.0b013e31829f5c58 (2013). 23958816

[b44] Organisation N. A. C. (ed Ministry of Health & Family Welfare) (Government of India, 2015).

[b45] WHO. WHO Child Growth Standards. Report No. ISBN 92 4 154693 X, (Department of Nutrition for Health and Development, World Health Organization. http://www.who.int/childgrowth/standards/Technical_report.pdf, 2006).

[b46] Dhanasekaran S. . Identification of biomarkers for Mycobacterium tuberculosis infection and disease in BCG-vaccinated young children in Southern India. Genes and immunity 14, 356–364, doi: 10.1038/gene.2013.26 (2013). 23676757

[b47] Albert P. S. Modeling longitudinal biomarker data from multiple assays that have different known detection limits. Biometrics 64, 527–537, doi: 10.1111/j.1541-0420.2007.00886.x (2008). 17764483

