# Supplementary material for: BLR1 and FCGR1A transcripts in peripheral blood associate with the extent of intrathoracic tuberculosis in children and predict treatment outcome
Source: Sci Rep. 2016 Dec 12;6:38841. doi: 10.1038/srep38841 (PMC5150239; doi:10.1038/srep38841)
Supplement: Supplementary Tables [file srep38841-s1.pdf]

Title: BLR1 and FCGR1A transcripts in peripheral blood associate with the extent of intrathoracic tuberculosis in children and predict treatment outcome.

Authors: Synne Jenum*[1], Rasmus Bakken[ƚ2], Dhanasekaran S[ƚ2], Aparna Mukherjee[3], Rakesh Lodha[3], Sarman Singh[4], Varinder Singh[5], Marielle C Haks[6], Tom HM Ottenhoff[6], SK Kabra[3], T Mark Doherty[7], Christian Ritz[8] and Harleen MS Grewal*[2,2a].

Authors' Addresses: [1]*Department of Clinical Science, Faculty of Medicine and Dentistry, University of Bergen, and Department of Infectious Diseases, Oslo University Hospital, Oslo, Norway.* [2]*Department of Clinical Science, Faculty of Medicine and Dentistry, University of Bergen, Norway.* [2a]*Department of Microbiology, Haukeland university hospital, University of Bergen, N-5021, Norway.* [3]*Department of Pediatrics, All India Institute of Medical Sciences, New Delhi, India.* [4]*Division of Clinical Microbiology & Molecular Medicine, Department of Laboratory Medicine, All India Institute of Medical Sciences, New Delhi, India.* [5]*Department of Pediatrics, Kalawati Saran Children Hospital, New Delhi, India.* [6]*Department of Infectious Diseases Group, Immunology and Immunogenetics of Bacterial Infectious Disease, Leiden University Medical Center, The Netherland.* [7]*GlaxoSmithKline Vaccines, Warve, Belgium.* [8]*Department of Nutrition, Exercise and Sports, University of Copenhagen, Denmark.*

[ƚ]Equal contribution

***CORRESPONDING AUTHORS**

Harleen M.S Grewal MD, PhD, DTMH

Professor and Senior Consultant,
Department of Clinical science infection, Faculty of Medicine and Dentistry
University of Bergen & Department of Microbiology
Haukeland University Hospital
Bergen 5021, Norway.
Phone: +47 55-97-4631; Mobile: +47 99450554; Fax: +47 55-97-4689
Email- Harleen.Grewal@k2.uib.no

Synne Jenum MD, PhD

Researcher and Medical Consultant
Department of Clinical science infection, Faculty of Medicine and Dentistry
University of Bergen
Bergen 5021, Norway, &
Department of Infectious Diseases, Oslo University Hospital, Oslo 0424, Norway
Mobile: +47 92249736
Email: synnejenum@gmail.com

Key words (MeSH terms):

Pulmonary tuberculosis; children; host biomarkers; Mycobacterium tuberculosis, treatment response.

Supplementary Table 1. Transcriptional markers measured direct ex-vivo showing significant fold change from baseline to 2 months on treatment in the four micronutrient intervention groups **(A)**. Proteomic markers measured direct ex-vivo showing significant fold change from baseline to 2 **(B)** and 6 months **(C)** on treatment in the four micronutrient intervention groups. Blank field means no significant change. 95% confidence intervals are given in parentheses.

A

	MN+Zn	MN	Zn	Placebo
N baseline	23	18	23	24
N 2 months	13	15	18	19
ABR			1,23 (1,03-1,47)	
B2M		0,69 (0,60-0,79)	0,77 (0,68-0,88)	
BCL2	1,79 (1,36-2,34)	1,77 (1,36-2,29)	1,54 (1,22-1,94)	1,9 (1,51-2,38)
BLR1	2,63 (1,60-4,31)	1,94 (1,20-3,14)	2,26 (1,47-3,46)	1,97 (1,29-3,00)
CASP8	1,46 (1,21-1,76)	1,11 (0,93-1,33)	1,21 (1,03-1,42)	1,25 (1,07-1,47)
CCR7	1,56 (1,14-2,13)		2,09 (1,59-2,74)	1,32 (1,01-1,72)
CD14	0,41 (0,28-0,59)			
CD19	1,74 (1,22-2,48)	1,47 (1,00-2,16)	2,4 (1,73-3,33)	2,41 (1,75-3,30)
CD3E	1,93 (1,41-2,64)	2,01 (1,48-2,72)	1,76 (1,34-2,31)	1,96 (1,5-2,56)
CD4	1,46 (1,04-2,05)	1,43 (1,04-1,99)	1,79 (1,34-2,41)	1,47 (1,1-1,96)
CD8A				0,39 (0,15-0,99)
FCGR1A	0,43 (0,29-0,63)	0,51 (0,35-0,75)	0,52 (0,37-0,72)	0,71 (0,51-0,99)
FOXP3		0,009 (0,00-0,94)	0,004 (0,00-0,39)	
FPR1	0,63 (0,51-0,78)	0,74 (0,60-0,91)	0,78 (0,65-0,93)	0,76 (0,63-0,91)
GUSB		1,35 (1,07-1,70)	1,58 (1,29-1,95)	1,23 (1,00-1,51)
IL7R	1,83 (1,41-2,36)	1,54 (1,20-1,99)	1,46 (1,17-1,82)	1,57 (1,26-1,96)
LTF				0,46 (0,33-0,64)
MARCO				0,69 (0,49-0,96)
MMP9	0,25 (0,14-0,45)	0,55 (0,32-0,93)	0,44 (0,26-0,74)	0,22 (0,13-0,37)
NCAM1			1,74 (1,30-2,32)	
RAB24	0,68 (0,47-0,96)	0,71 (0,51-0,99)		
SEC14L1			1,28 (1,01-1,62)	
TGFB1			1,23 (1,02-1,48)	
TGFBR2	1,51 (1,26-1,81)	1,26 (1,06-1,50)	1,38 (1,18-1,62)	1,2 (1,03-1,40)
TIMP2	0,75 (0,65-0,87)			
TNFRSF1B			1,72 (1,27-2,33)	

B

	MN+Zn	MN	Zn	Placebo
N baseline	23	18	23	24
N 2 months*	13	15	18	19
IFNα-2	0,57 (0,38-0,85)	0,57 (0,40-0,82)	1,41 (1,04-0,82)	
IFNγ		0,46 (0,28-0,73)	0,48 (0,32-0,73)	0,38 (0,25-0,6)
IL12p70		0,49 (0,25-0,98)		
IL-13	4,46 (2,03-9,81)		2,10 (1,02-4,32)	
IL-17A		0,54 (0,32-0,93)	0,73 (0,54-0,99)	
IL-1β			0,35 (0,16-0,74)	
IL-2		0,40 (0,20-0,78)	0,37 (0,21-0,66)	0,39 (0,21-0,72)
IL-5			2,07 (1,21-3,52)	
IP-10			0,69 (0,53-0,91)	
MCP3	2,73 (1,35-5,50)			

C

	Placebo
N baseline	24
N 6 months	10
IFNα-2	
IFNγ	0,38 (0,24-0,62)
IL12p70	
IL-13	
IL-17A	0,34 (0,21-0,56)
IL-1β	
IL-2	0,30 (0,14-0,65)
IL-5	
IP-10	0,59 (0,43-0,80)
MCP3	

% fold decrease	
	≤30
	>30, ≤50
	>50, ≤70
	>70, ≤90
	>90, ≤200
	>200

% fold increase	
	≤30
	>30, ≤50
	>50, ≤70
	>70, ≤90
	>90, ≤200
	>200

Supplementary Table 2: Genes investigated in the dcRT-MLPA and their functions. The table has been modified from table previously published by the same authors.[1]

Genes	Putative role	Reference	Method for biomarker discovery[2]
**BCL2**	B-cell lymphoma 2, down-regulates cell death (apoptosis). Increased *BCL2* expression in infected macrophages may create an immune privileged site for MTB infection and is implicated in poor prognosis for TB patients.	[3,4]	A, C
**BLR1**	Belongs to CXC chemokine receptor family (CXCR5), located in lymph node follicles. An increased expression of *BLR1* in TB patients might help in sustaining the expression of its ligand CXCL13, which in turn attracts B cells.	[5]	C
**BPI**	Bactericidal permeability-increasing protein, found in neutrophil granules and associated with host defence through microbiocidal activity.	[6]	C
**CASP8**	Cysteine-aspartic acid protease family, plays a central role in the execution-phase of cell apoptosis and increased expression is seen in TB patients	[7,8]	C
**CCL4**	Chemokine (c-c motif) ligand 4 or Macrophage inflammatory protein 1β (MIP-1β), acts as chemo-attractant for a variety of immune cells.	[9]	C
**CCL13**	Chemokine (c-c motif) ligand 13, link immune activation to the recruitment of leukocytes in both Th1-and Th2-type immune responses.	[10]	B,C
**CCL19**	Chemokine (c-c motif) ligand 19, contributes to control of MTB infection through homing of lymphocytes and dendritic cells.	[11]	B
**CCL22**	Belongs to CC chemokine family, plays a role in the trafficking of activated T cells to inflammatory sites.	[9]	C
**CCR7**	CC chemokine receptor 7, mediates trafficking of dendritic cells and T cells from the lungs to the mediastinal lymph node during TB disease.	[12,13]	C
**CD3E**	T-cell glycoprotein CD3 epsilon, plays a role in intracellular signal-transduction pathways.	[14]	C
**CD4**	Cluster of differentiation 4, plays a critical role in adaptive immunity to TB.	[2]	C
**CD8A**	Cluster of differentiation 8A, identifies cytotoxic T- cells that interact with MHC class I targets and may contribute to control of MTB infection.	[15]	C
**CD14**	Cluster of differentiation 14, a component of the innate immune system and acts a co-receptor for recognizing MTB. Monocyte/macrophage marker.	[16,17]	C
**CD19**	Cluster of differentiation 19, a B cell marker and essential for B cell activation.	[18]	C
**CD163**	Cluster of differentiation 163, a hemoglobin scavenger receptor exclusively expressed in macrophages upon inflammation.	[19]	C
**CTLA4**	Cytotoxic T-lymphocyte antigen 4 plays a role in the down-regulation of T cell immune responses and involved in the maintenance of T cell homeostasis. *CTLA4* gene expression may be associated with severity of pulmonary TB.	[20,21]	B,C
**CXCL10**	CXC chemokine 10 or interferon gamma-induced protein 10 (IP-10), secreted by number of cell types in response to IFN-α/β and involved in stimulation of natural killer cells and T-cell migration in MTB infection.	[22]	A, C
**FASLG**	Fas ligand, a type II transmembrane protein, belonging to the TNF family involved in immune modulation and pathogenesis of TB. Modulators of Fas/FasL-mediated apoptosis may therefore be clinically useful.	[23]	C

**FCGR1A**	The Fc region of immunoglobulin gamma is involved in both innate and adaptive immune responses. It aids to control MTB infection and plays a central role in antibody-dependent cytotoxicity and the clearance of immune complexes.	24	C
**FOXP3**	Forkhead box P3, belongs to the transcription factor family and is involved in various cellular processes, acts as an important regulator for T-cell development.	25	C
**FPR1**	Formyl peptide receptor 1, a member of G-protein-coupled receptor family involved in the control of inflammation and neutrophil function.	26	C
**IFNγ**	Interferon gamma, secreted by several cell types such as NK cells, $CD4^+$ and $CD8^+$ T-cells, essential for control of MTB infection.	27	B
**IL2RA**	Interleukin 2 receptor gene, plays an important role in the activation and expansion of T-cells.	28	C
**IL4**	Interleukin 4, the prototypical Th2 cytokine; high levels of expression are associated with poor outcomes in TB.	29	C
**IL4d2**	IL-4 antagonist and splice variant of interleukin-4.	30	C
**IL7R**	Interleukin-7 receptor, plays a role in the development of immune cells and control of apoptosis.	31	C
**IL10**	Interleukin-10, an anti-inflammatory cytokine that may contribute to TB pathogenesis. IL-10 blocks phagosome maturation by a STAT3-dependent, p38-independent mechanism, which facilitates MTB survival and outgrowth	32	A
**IL22RA1**	Interleukin 22 receptor alpha 1belongs to class II cytokine receptor family and activates various signaling pathways. The expression of *IL22RA1* was found to be higher in late TB granulomas.	33,34	B
**LAG3**	Lymphocyte-activation gene 3, an important regulatory molecule involved in controlling the expansion and activation of T-cells.	35	A,C
**LTF**	Lacto-transferrin also called Lactoferrin (LF), a secreted mediator that connects innate and adaptive immune responses.	36	C
**MARCO**	Antigen-presenting cell scavenger receptor, involved in TLR activation that mediates phagocytosis of pathogens.	37	C
**MMP9**	Matrix metallo-peptidase 9, induced by MTB infection and has a role together with MCP-1in recruiting macrophages to the lungs during granuloma formation.	38	C
**NCAM1**	Neural cell adhesion molecule 1, mediates several intracellular signaling pathways and may be involved in TB pathogenesis.	39,40	A
**RAB13**	Ras related protein-13, a small GTPase family member, regulates assembly of functional tight junctions in epithelial cells.	41	C
**RAB24**	Ras related protein-24, a small GTPase family member, regulates intracellular protein trafficking between endoplasmic reticulum and cis-Golgi compartment.	41	C
**RAB33A**	Ras related protein-33A, a small GTPase family member; Dysregulation of GTPase plays a role in blocking of phagosome maturation, which is a major survival strategy for MTB.	42	C
**SEC14L1**	SEC14 cytosolic factor family plays a role in lipid metabolism and the intracellular transport system. SEC14L1 also appears to be a negative regulator of some innate immune functions.	43,44	C
**SPP1**	Secreted phosphoprotein 1, acts as a cytokine that up-regulates the expression of interferon-gamma and interleukin-12, contributes to resistance against mycobacteria by boosting reactive oxygen intermediate production in macrophages.	45,46	C
**TGFB1**	Transforming growth factor β1, an anti-inflammatory cytokine, performs many cellular functions and is involved in resolution of granulomatous lesions in TB.	47	C

**TGFBR2**	Transforming growth factor β receptor 2, involved in signal transduction and response to inhibit cell growth and division.	[48]	C
**TIMP2**	Tissue inhibitor of metallo-proteinases, involved in pathological changes, tissue remodeling and possibly, pathogenesis of pulmonary TB.	[49]	C
**TNF**	Tumor necrosis factor, a cytokine that plays multiple roles in the immunopathology of TB and is essential for controlling MTB infection.	[50]	B, C
**TNFRSF1A**	TNF receptor superfamily member1A, mediates apoptosis and functions as a regulator of inflammation.	[51]	C
**TNFRSF1B**	TNF receptor superfamily member 1B, mediates anti-apoptotic signals.	[51]	C
**TNFRSF18**	TNF receptor superfamily member 18, involved in T-cell activation, programmed cell death and pulmonary fibrosis.	[49,51]	C
**ABR**	Active BCR-Related gene contains a GTPase-activating protein domain (used as endogenous control).		
**B2M**	β2 microglobulin, a component of MHC class I molecules (used as endogenous control).		C
**GAPDH**	Glyceraldehyde 3-phosphate dehydrogenase, involved in quite a few non-metabolic processes (used as endogenous control).		
**GUSB**	Glucuronidase β, regulates lysosomal storage function and co-regulated in response to stress (used as endogenous control).	[52]	

(A) Experimental in vitro studies, (B) Experimental in vivo studies, (C) ELISA/ELISPOT/Microarray/qPCR on clinical samples.

References for Supplementary Table 1

1 Jenum, S. *et al.* Approaching a diagnostic point-of-care test for pediatric tuberculosis through evaluation of immune biomarkers across the clinical disease spectrum. *Sci Rep* **6**, 18520, doi:10.1038/srep18520 (2016).

2 Joosten, S. A. *et al.* Identification of biomarkers for tuberculosis disease using a novel dual-color RT-MLPA assay. *Genes and immunity* **13**, 71-82, doi:10.1038/gene.2011.64 (2012).

3 Mogga, S. J., Mustafa, T., Sviland, L. & Nilsen, R. Increased Bcl-2 and reduced Bax expression in infected macrophages in slowly progressive primary murine Mycobacterium tuberculosis infection. *Scand J Immunol* **56**, 383-391 (2002).

4 Elliott, T. O. *et al.* Dysregulation of Apoptosis Is a Risk Factor for Tuberculosis Disease Progression. *J Infect Dis*, doi:10.1093/infdis/jiv238 (2015).

5 Mihret, A. *et al.* Combination of gene expression patterns in whole blood discriminate between tuberculosis infection states. *BMC Infect Dis* **14**, 257, doi:10.1186/1471-2334-14-257 (2014).

6 Schultz, H. & Weiss, J. P. The bactericidal/permeability-increasing protein (BPI) in infection and inflammatory disease. *Clinica chimica acta; international journal of clinical chemistry* **384**, 12-23, doi:10.1016/j.cca.2007.07.005 (2007).

7 Elmore, S. Apoptosis: a review of programmed cell death. *Toxicologic pathology* **35**, 495-516, doi:10.1080/01926230701320337 (2007).

8 Sloot, R. *et al.* Biomarkers Can Identify Pulmonary Tuberculosis in HIV-infected Drug Users Months Prior to Clinical Diagnosis. *EBioMedicine* **2**, 172-179, doi:10.1016/j.ebiom.2014.12.001 (2015).

9 Le, Y., Zhou, Y., Iribarren, P. & Wang, J. Chemokines and chemokine receptors: their manifold roles in homeostasis and disease. *Cellular & molecular immunology* **1**, 95-104 (2004).

10 Garcia-Zepeda, E. A. *et al.* Human monocyte chemoattractant protein (MCP)-4 is a novel CC chemokine with activities on monocytes, eosinophils, and basophils induced in allergic and nonallergic inflammation that signals through the CC chemokine receptors (CCR)-2 and -3. *Journal of immunology* **157**, 5613-5626 (1996).

11 Khader, S. A. *et al.* In a murine tuberculosis model, the absence of homeostatic chemokines delays granuloma formation and protective immunity. *Journal of immunology* **183**, 8004-8014, doi:10.4049/jimmunol.0901937 (2009).

12 Olmos, S., Stukes, S. & Ernst, J. D. Ectopic activation of Mycobacterium tuberculosis-specific CD4+ T cells in lungs of CCR7-/- mice. *Journal of immunology* **184**, 895-901, doi:10.4049/jimmunol.0901230 (2010).

13 Haynes, N. M. *et al.* Role of CXCR5 and CCR7 in follicular Th cell positioning and appearance of a programmed cell death gene-1high germinal center-associated subpopulation. *Journal of immunology* **179**, 5099-5108 (2007).

14 Banner, B., Spicer, Z. & Alroy, J. Expression of CD3 epsilon subunit in gastric parietal cells: a possible role in signal transduction? *Pathology, research and practice* **199**, 137-143 (2003).

15 Lin, P. L. & Flynn, J. L. CD8 T cells and Mycobacterium tuberculosis infection. *Semin Immunopathol* **37**, 239-249, doi:10.1007/s00281-015-0490-8 (2015).

16 Wang, C. *et al.* Serum complement C4b, fibronectin, and prolidase are associated with the pathological changes of pulmonary tuberculosis. *BMC Infect Dis* **14**, 52, doi:10.1186/1471-2334-14-52 (2014).

17 Ayaslioglu, E. *et al.* The role of CD14 gene promoter polymorphism in tuberculosis susceptibility. *J Microbiol Immunol Infect* **46**, 158-163, doi:10.1016/j.jmii.2012.05.008 (2013).

18 Depoil, D. *et al.* CD19 is essential for B cell activation by promoting B cell receptor-antigen microcluster formation in response to membrane-bound ligand. *Nature immunology* **9**, 63-72, doi:10.1038/ni1547 (2008).

19 Moestrup, S. K. & Moller, H. J. CD163: a regulated hemoglobin scavenger receptor with a role in the anti-inflammatory response. *Annals of medicine* **36**, 347-354 (2004).

20 McCoy, K. D. & Le Gros, G. The role of CTLA-4 in the regulation of T cell immune responses. *Immunology and cell biology* **77**, 1-10, doi:10.1046/j.1440-1711.1999.00795.x (1999).

21 Wang, C. *et al.* Association of CTLA4 gene polymorphisms with susceptibility and pathology correlation to pulmonary tuberculosis in Southern Han Chinese. *Int J Biol Sci* **8**, 945-952, doi:10.7150/ijbs.4390 (2012).

22 Lande, R. *et al.* IFN-alpha beta released by Mycobacterium tuberculosis-infected human dendritic cells induces the expression of CXCL10: selective recruitment of NK and activated T cells. *Journal of immunology* **170**, 1174-1182 (2003).

23 Mustafa, T., Mogga, S. J., Mfinanga, S. G., Morkve, O. & Sviland, L. Significance of Fas and Fas ligand in tuberculous lymphadenitis. *Immunology* **114**, 255-262, doi:10.1111/j.1365-2567.2004.02080.x (2005).

24 Sutherland, J. S. *et al.* Differential gene expression of activating Fcgamma receptor classifies active tuberculosis regardless of human immunodeficiency virus status or ethnicity. *Clin Microbiol Infect* **20**, O230-238, doi:10.1111/1469-0691.12383 (2014).

25 Larson, R. P., Shafiani, S. & Urdahl, K. B. Foxp3(+) regulatory T cells in tuberculosis. *Adv Exp Med Biol* **783**, 165-180, doi:10.1007/978-1-4614-6111-1_9 (2013).

26 Dorward, D. A. *et al.* The role of formylated peptides and formyl peptide receptor 1 in governing neutrophil function during acute inflammation. *Am J Pathol* **185**, 1172-1184, doi:10.1016/j.ajpath.2015.01.020 (2015).

27 Fenton, M. J. *et al.* Induction of gamma interferon production in human alveolar macrophages by Mycobacterium tuberculosis. *Infect Immun* **65**, 5149-5156 (1997).

28 Milani, P. *et al.* Mechanics of the IL2RA gene activation revealed by modeling and atomic force microscopy. *PloS one* **6**, e18811, doi:10.1371/journal.pone.0018811 (2011).

29 Ashenafi, S. *et al.* Progression of clinical tuberculosis is associated with a Th2 immune response signature in combination with elevated levels of SOCS3. *Clin Immunol* **151**, 84-99, doi:10.1016/j.clim.2014.01.010 (2014).

30 Wassie, L. *et al.* Ex vivo cytokine mRNA levels correlate with changing clinical status of ethiopian TB patients and their contacts over time. *PloS one* **3**, e1522, doi:10.1371/journal.pone.0001522 (2008).

31 Akashi, K., Kondo, M. & Weissman, I. L. Role of interleukin-7 in T-cell development from hematopoietic stem cells. *Immunological reviews* **165**, 13-28 (1998).

32 O'Leary, S., O'Sullivan, M. P. & Keane, J. IL-10 blocks phagosome maturation in mycobacterium tuberculosis-infected human macrophages. *Am J Respir Cell Mol Biol* **45**, 172-180, doi:10.1165/rcmb.2010-0319OC (2011).

33 Mehra, S. *et al.* Transcriptional reprogramming in nonhuman primate (rhesus macaque) tuberculosis granulomas. *PloS one* **5**, e12266, doi:10.1371/journal.pone.0012266 (2010).

34 Lim, C. & Savan, R. The role of the IL-22/IL-22R1 axis in cancer. *Cytokine Growth Factor Rev* **25**, 257-271, doi:10.1016/j.cytogfr.2014.04.005 (2014).
35 Sierro, S., Romero, P. & Speiser, D. E. The CD4-like molecule LAG-3, biology and therapeutic applications. *Expert Opin Ther Targets* **15**, 91-101, doi:10.1517/14712598.2011.540563 (2011).
36 Siqueiros-Cendon, T. *et al.* Immunomodulatory effects of lactoferrin. *Acta pharmacologica Sinica* **35**, 557-566, doi:10.1038/aps.2013.200 (2014).
37 Komine, H., Kuhn, L., Matsushita, N., Mule, J. J. & Pilon-Thomas, S. Examination of MARCO activity on dendritic cell phenotype and function using a gene knockout mouse. *PloS one* **8**, e67795, doi:10.1371/journal.pone.0067795 (2013).
38 Taylor, J. L. *et al.* Role for matrix metalloproteinase 9 in granuloma formation during pulmonary Mycobacterium tuberculosis infection. *Infect Immun* **74**, 6135-6144, doi:10.1128/IAI.02048-05 (2006).
39 Hortsch, M. & Umemori, H. *The sticky synapse: cell adhesion molecules and their role in synapse formation and maintenance*. (Springer, 2009).
40 Rivera-Marrero, C. A., Stewart, J., Shafer, W. M. & Roman, J. The down-regulation of cathepsin G in THP-1 monocytes after infection with Mycobacterium tuberculosis is associated with increased intracellular survival of bacilli. *Infect Immun* **72**, 5712-5721, doi:10.1128/IAI.72.10.5712-5721.2004 (2004).
41 Jacobsen, M. *et al.* Ras-associated small GTPase 33A, a novel T cell factor, is down-regulated in patients with tuberculosis. *J Infect Dis* **192**, 1211-1218, doi:10.1086/444428 (2005).
42 Doherty, M., Wallis, R. S., Zumla, A. & group, W. H.-T. D. R. E. C. j. e. c. Biomarkers for tuberculosis disease status and diagnosis. *Current opinion in pulmonary medicine* **15**, 181-187 (2009).
43 Mousley, C. J., Tyeryar, K. R., Vincent-Pope, P. & Bankaitis, V. A. The Sec14-superfamily and the regulatory interface between phospholipid metabolism and membrane trafficking. *Biochim Biophys Acta* **1771**, 727-736, doi:10.1016/j.bbalip.2007.04.002 (2007).
44 Li, M. T. *et al.* Negative regulation of RIG-I-mediated innate antiviral signaling by SEC14L1. *J Virol* **87**, 10037-10046, doi:10.1128/JVI.01073-13 (2013).
45 Renkl, A. C. *et al.* Osteopontin functionally activates dendritic cells and induces their differentiation toward a Th1-polarizing phenotype. *Blood* **106**, 946-955, doi:10.1182/blood-2004-08-3228 (2005).
46 Khajoee, V. *et al.* Novel roles of osteopontin and CXC chemokine ligand 7 in the defence against mycobacterial infection. *Clin Exp Immunol* **143**, 260-268, doi:10.1111/j.1365-2249.2005.02985.x (2006).
47 Toossi, Z., Gogate, P., Shiratsuchi, H., Young, T. & Ellner, J. J. Enhanced production of TGF-beta by blood monocytes from patients with active tuberculosis and presence of TGF-beta in tuberculous granulomatous lung lesions. *Journal of immunology* **154**, 465-473 (1995).
48 Bellam, N. & Pasche, B. Tgf-beta signaling alterations and colon cancer. *Cancer treatment and research* **155**, 85-103, doi:10.1007/978-1-4419-6033-7_5 (2010).
49 Zhang, Q., Guo, Y., Dong, R., Dai, R. & Zhou, M. Suppressor of cytokine signaling 1-modulated metalloproteinases and tissue inhibitor of metalloproteinase in pulmonary fibrosis. *Mol Med Rep* **12**, 3855-3861, doi:10.3892/mmr.2015.3810 (2015).
50 Lin, P. L., Plessner, H. L., Voitenok, N. N. & Flynn, J. L. Tumor necrosis factor and tuberculosis. *J Investig Dermatol Symp Proc* **12**, 22-25, doi:10.1038/sj.jidsymp.5650027 (2007).
51 Croft, M. The role of TNF superfamily members in T-cell function and diseases. *Nature reviews. Immunology* **9**, 271-285, doi:10.1038/nri2526 (2009).
52 Dhanasekaran, S. *et al.* Identification of biomarkers for Mycobacterium tuberculosis infection and disease in BCG-vaccinated young children in Southern India. *Genes and immunity* **14**, 356-364, doi:10.1038/gene.2013.26 (2013).
